# Quercetin Alleviates All-*Trans*-Retinal-Induced Photoreceptor Apoptosis and Retinal Degeneration by Inhibiting the ER Stress-Related PERK Signaling

**DOI:** 10.3390/ijms252413624

**Published:** 2024-12-19

**Authors:** Bo Yang, Kunhuan Yang, Ruitong Xi, Jingmeng Chen, Yalin Wu

**Affiliations:** 1 Fujian Provincial Key Laboratory of Ophthalmology and Visual Science, Fujian Engineering and Research Center of Eye Regenerative Medicine, Eye Institute of Xiamen University, School of Medicine, Xiamen University, Xiamen 361102, China; 2 School of Medicine, Xiamen University, Xiamen 361102, China; 3 Shenzhen Research Institute of Xiamen University, Shenzhen 518057, China

**Keywords:** quercetin, photoreceptor, all-*trans*-retinal, ER stress, apoptosis

## Abstract

All-*trans*-retinal (atRAL)-induced photoreceptor atrophy and retinal degeneration are hallmark features of dry age-related macular degeneration (AMD) and Stargardt disease type 1 (STGD1). The toxicity of atRAL is closely related to the generation of reactive oxygen species (ROS). Quercetin, a natural product, is known for its potent antioxidant properties; however, its effects in mitigating atRAL-mediated retinal damage remains unclear. This study investigated the protective effects of quercetin against atRAL-induced photoreceptor damage. Using atRAL-loaded 661W photoreceptor cells, we evaluated cell viability, ROS generation, and endoplasmic reticulum (ER) stress under quercetin treatment. Quercetin significantly restored the cell viability (to 70%) and reduced ROS generation in atRAL-treated 661W cells. Additionally, Western blot analysis demonstrated that quercetin mitigated protein kinase RNA-like ER kinase (PERK) signaling, preventing ER stress-induced apoptosis. Importantly, in *Abca4^−/−^Rdh8^−/−^* mice, an animal model of light-induced atRAL accumulation in the retina, quercetin treatment effectively alleviated light-exposed photoreceptor atrophy and retinal degeneration by attenuating PERK signaling. Thus, quercetin protected photoreceptor cells from atRAL-induced damage by inhibiting ROS generation and PERK signaling, which suggests its potential as a therapeutic agent for atRAL-related retinal degeneration.

## 1. Introduction

Age-related macular degeneration (AMD), one of the leading causes of blindness among the elderly in developed countries, is projected to affect over 288 million individuals by 2040 [1,2]. Dry AMD is the predominant form, accounting for 85−90% of AMD cases [3]. Similarly, Stargardt disease type 1 (STGD1) is recognized as the most common form of juvenile macular degeneration [4]. Photoreceptor atrophy is a significant pathological hallmark shared by both dry AMD and STGD1, which is closely associated with the accumulation of all-*trans*-retinal (atRAL) due to disruptions in the visual (retinoid) cycle [5,6]. Accumulation of atRAL promotes the production of reactive oxygen species (ROS), leading to oxidative stress—a critical factor in photoreceptor damage [7]. ATP-binding cassette transporter 4 (ABCA4) and retinol dehydrogenase 8 (RDH8) are key proteins involved in atRAL clearance within the visual cycle, and their dysfunction exacerbates atRAL accumulation [8,9]. Mice lacking ABCA4 and RDH8 (*Abca4^−/−^Rdh8^−/−^* mice) exhibit impaired atRAL clearance and develop photoreceptor atrophy with age, mirroring the pathological phenotype observed in dry AMD and STGD1 patients [10,11]. Exposure to bright light accelerates visual cycle disruption in *Abca4^−/−^Rdh8^−/−^* mice, resulting in atRAL-induced photoreceptor atrophy and retinal degeneration [7].

Endoplasmic reticulum (ER) stress is a cellular condition characterized by the accumulation of misfolded or unfolded proteins within the ER lumen, which disrupts ER homeostasis and activates a series of signaling pathways known as the unfolded protein response (UPR) [12,13]. The protein kinase RNA-like ER kinase (PERK) pathway is a major branch of the UPR activated in response to ER stress [14,15]. The UPR facilitates the dissociation of binding immunoglobulin protein (BiP) from PERK, resulting in PERK activation through oligomerization and autophosphorylation [16,17]. Activated PERK (p-PERK) phosphorylates eukaryotic initiation factor 2α (eIF2α), resulting in a global reduction in protein synthesis to alleviate the burden on the ER [18,19]. However, phosphorylated eIF2α (p-eIF2α) selectively enhances the translation of activating transcription factor 4 (ATF4) [20,21]. ATF4 subsequently upregulates genes involved in amino acid metabolism, redox homeostasis, and apoptosis, thus contributing to cellular adaptation during ER stress [22,23]. If ER stress remains unresolved, prolonged ATF4 activation induces pro-apoptotic factors such as C/EBP homologous protein (CHOP), ultimately leading to cell death [24,25]. Recent studies have shown that atRAL induces photoreceptor apoptosis through activation of the PERK/eIF2α/ATF4/CHOP pathway during ER stress [26]. Therefore, the PERK pathway in ER stress may be a potential target for atRAL-related retinopathy.

Dietary polyphenols are a diverse class of natural compounds known for their extensive biological activities, including potent antioxidant, anti-inflammatory, and anti-apoptotic effects [27,28]. Among these, quercetin stands out due to its accessibility from dietary sources and superior efficacy [29]. Notably, comparative analyses of three dietary polyphenols (delphinidin, resveratrol, and quercetin) have demonstrated that quercetin exhibits the strongest antioxidant properties [30]. As a polyphenolic compound, quercetin exerts its biological effects primarily by reducing ROS generation [31,32]. Recently, Feng et al. demonstrated that quercetin mitigates chondrocyte apoptosis in osteoarthritis, which is closely associated with reduced oxidative stress [33]. Furthermore, Sang et al. reported that quercetin alleviates acute lung injury by inhibiting oxidative stress-mediated ER stress [34]. However, the protective effect of quercetin on atRAL-induced photoreceptor damage remains unclear. This study aims to investigate the impact of quercetin on the PERK/eIF2α/ATF4/CHOP pathway in oxidative stress-mediated ER stress and to assess its therapeutic efficacy in photoreceptors exposed to atRAL.

## 2. Results

### 2.1. Quercetin Attenuates the Toxicity of atRAL in 661W Cells by Decreasing ROS Generation

**Figure 1:**
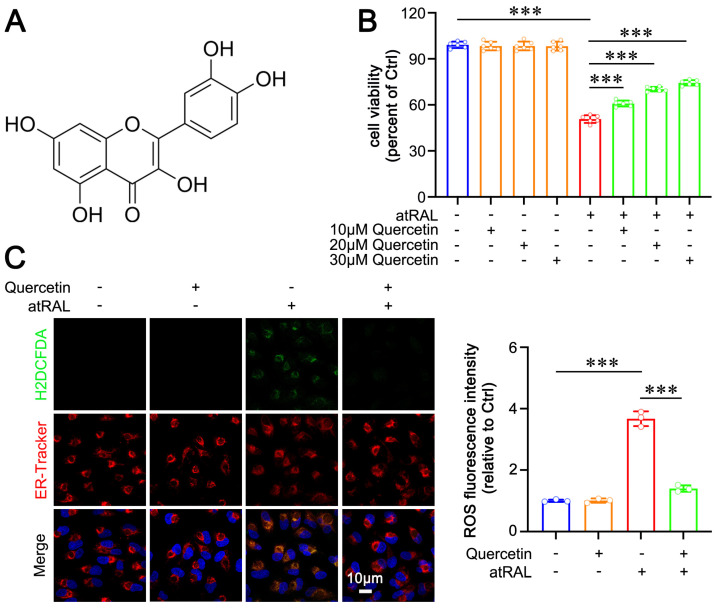
Quercetin restores cell viability and suppresses ROS generation in atRAL-treated 661W cells. (**A**) Structure of quercetin. (**B**) Cell viability (n = 6). (**C**) Fluorescence imaging of ROS and ER (n = 3). Scale bars, 10 μm. *** *p* < 0.001.

Quercetin is a hydroxyl-rich compound (Figure 1A) with potent antioxidant properties [35]. We evaluated its effects on atRAL toxicity in the mouse photoreceptor cell line 661W. Liao et al. established a model of 5 µM atRAL-induced cytotoxicity in 661W photoreceptor cells [7]. The impact of quercetin on cell viability in 5 µM atRAL-treated 661W cells was analyzed using the MTS assay. The results demonstrated that treatment with gradient concentrations of quercetin (10, 20, and 30 µM) significantly restored the viability of 661W cells exposed to atRAL in a concentration-dependent manner (Figure 1B). Consequently, a concentration of 20 µM quercetin was utilized in subsequent experiments. Our prior studies have confirmed that atRAL induces ROS generation to trigger ER stress [26]. In this context, we investigated the effects of quercetin on ROS generation and its localization within the ER. Co-staining with the H2DCFDA probe and an ER-Tracker probe revealed that quercetin significantly attenuated both ROS production and its localization within the ER (Figure 1C). These findings indicate that quercetin effectively mitigates atRAL-induced damage and ROS generation in photoreceptor cells.

### 2.2. Quercetin Inhibits ER Stress-Mediated PERK/eIF2α/ATF4/CHOP Signaling Activation in atRAL-Loaded 661W Cells

**Figure 2:**
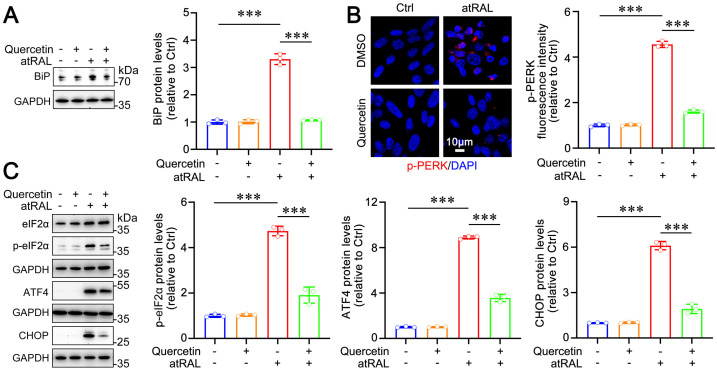
Effect of quercetin on the PERK/eIF2α/ATF4/CHOP signaling in atRAL-loaded cells. (**A**) Immunoblotting analysis and quantification of BiP (n = 3). (**B**) Immunofluorescence analysis and quantification of p-PERK (n = 3). Scale bars, 10 μm. (**C**) Immunoblotting analysis of eIF2α, p-eIF2α, ATF4, and CHOP, and quantification of p-eIF2α, ATF4, and CHOP (n = 3). *** *p* < 0.001.

Previous studies have demonstrated that atRAL induces ER stress by activating the PERK/eIF2α/ATF4/CHOP signaling pathway through oxidative stress [26]. Given quercetin’s ability to reduce the localization of ROS within the ER, we investigated its effects on this signaling cascade. Oxidative stress triggers the UPR in the ER, leading to the dissociation of binding BiP from PERK [16]. The freed PERK undergoes autophosphorylation, becoming activated [16]. Immunoblotting analyses revealed that quercetin significantly reduced the elevated levels of BiP protein induced by atRAL in 661W cells (Figure 2A). Immunofluorescence results indicated that quercetin inhibited the phosphorylation of PERK in atRAL-treated 661W cells (Figure 2B). The p-PERK subsequently phosphorylates eIF2α, which further activates ATF4 and CHOP, ultimately promoting cell apoptosis [36]. Immunoblotting results demonstrated that quercetin attenuated protein levels of phosphorylated eIF2α (p-eIF2α) and inhibited the expression of ATF4 and CHOP in 661W cells exposed to atRAL (Figure 2C). These findings suggest that quercetin effectively inactivates the PERK/eIF2α/ATF4/CHOP signaling pathway and prevents atRAL-induced ER stress in photoreceptor cells.

### 2.3. Quercetin Suppresses ER Stress-Induced Apoptosis in atRAL-Treated 661W Cells

**Figure 3:**
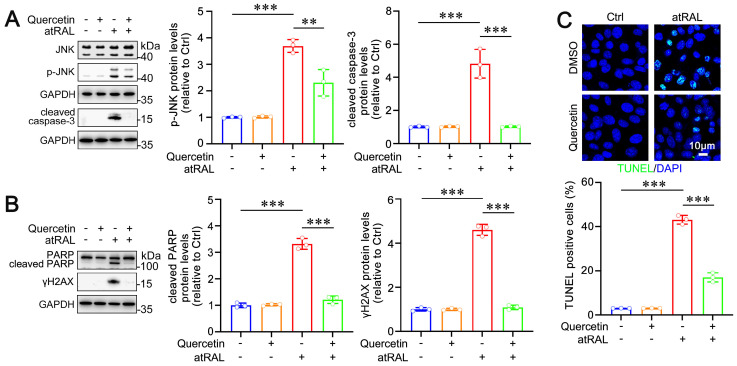
Effect of quercetin on atRAL-induced apoptosis in 661W cells. (**A**) Immunoblotting analysis of JNK, p-JNK, and cleaved caspase-3, and quantification of p-JNK and cleaved caspase-3 (n = 3). (**B**) Immunoblotting analysis of PARP, cleaved PARP, and *γ*H2AX, and quantification of cleaved PARP and *γ*H2AX (n = 3). (**C**) TUNEL staining (n = 3). Scale bars, 10 μm. ** *p* < 0.01 and *** *p* < 0.001.

As ER stress is a well-known trigger of apoptosis, we investigated the effect of quercetin on atRAL-induced apoptosis in photoreceptor cells. Our previous study has confirmed that atRAL promotes JNK phosphorylation (p-JNK) via p-eIF2α in 661W cells, with p-JNK subsequently inducing caspase-3 cleavage and promoting apoptosis [26]. Western blot analysis showed that quercetin significantly reduced protein levels of p-JNK and cleaved caspase-3 (Figure 3A). Moreover, the JNK-mediated DNA damage is also a contributor to apoptosis [7]. Quercetin markedly decreased protein levels of DNA damage markers, including cleaved PARP and γH2AX (Figure 3B). To further corroborate the anti-apoptotic effect of quercetin, we performed TUNEL staining, which revealed that quercetin visibly reduced the number of apoptotic 661W cells induced by atRAL (Figure 3C). These findings indicate that quercetin effectively mitigates atRAL-mediated apoptosis in photoreceptor cells by inhibiting ER stress-mediated JNK signaling.

### 2.4. Quercetin Alleviates Retinal Degeneration in Light-Exposed Abca4^−/−^Rdh8^−/−^ Mice

**Figure 4:**
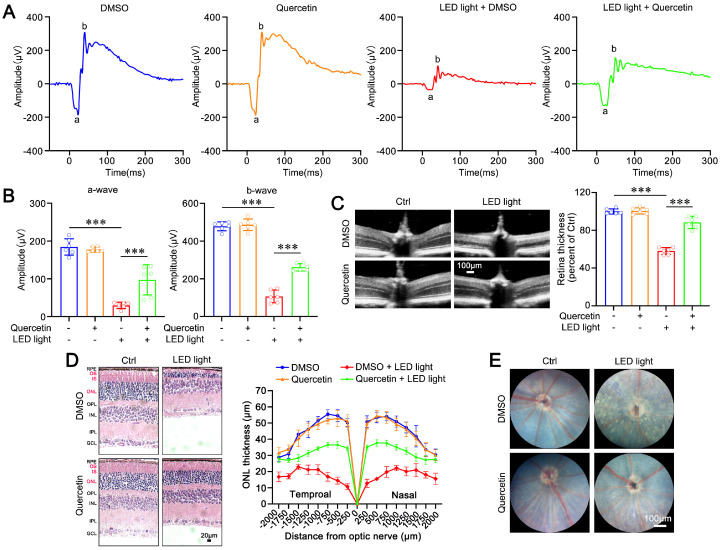
Effect of quercetin on the structure and function of the retina in light-exposed *Abca4^−/−^Rdh8^−/−^* mice. (**A**) Full-flash ERG. Stimulus luminance, 1 cd s/m^2^. a: the lowest point; b: the highest point (**B**) Quantification of full-flash ERG (n = 6). Stimulus luminance, 1 cd s/m^2^. (**C**) OCT imaging and quantification of retina thickness (n = 6). Scale bars, 100 μm. (**D**) H&E staining and quantification of ONL thickness (n = 6). Scale bars, 20 μm. (**E**) Fundus imaging. Scale bars, 100 μm. *** *p* < 0.001.

Given the protective effect of quercetin on photoreceptor cells against atRAL in vitro, we assessed its impact on light-induced retinal damage in *Abca4^−/−^Rdh8^−/−^* mice. Previous studies have shown that *Abca4^−/−^Rdh8^−/−^* mice develop obvious retinal degeneration after exposure to bright light compared with normal C57BL/6J mice [7]. Retinal function was evaluated using full-field ERG. It was found that quercetin dramatically prevented the decline in the amplitudes of both a-waves and b-waves in light-exposed *Abca4^−/−^Rdh8^−/−^* mice (Figure 4A,B). Photoreceptor atrophy was analyzed through OCT imaging and H&E staining. The results demonstrated that quercetin significantly restored the thickness of the total retinal layer and the outer nuclear layer (ONL) of *Abca4^−/−^Rdh8^−/−^* mice in response to light exposure (Figure 4C,D). Additionally, fundus imaging indicated that quercetin clearly alleviated light-induced punctate lesions in the retina of *Abca4^−/−^Rdh8^−/−^* mice (Figure 4E). Collectively, these findings suggest that quercetin treatment effectively mitigates both structural and functional damage to the retina induced by light in *Abca4^−/−^Rdh8^−/−^* mice.

### 2.5. Quercetin Ameliorates the PERK/eIF2α/ATF4/CHOP Signaling-Mediated Photoreceptor Apoptosis in Light-Exposed Abca4^−/−^Rdh8^−/−^ Mice

**Figure 5:**
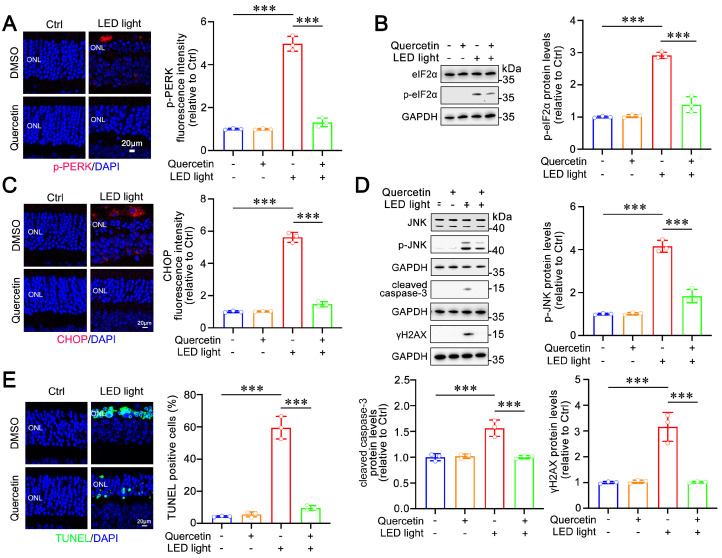
Effect of quercetin on the PERK/eIF2α/ATF4/CHOP signaling-induced photoreceptor apoptosis in light-exposed *Abca4^−/−^Rdh8^−/−^* mice. (**A**) Immunofluorescence analysis and quantification of p-PERK (n = 3). (**B**) Immunoblotting analysis of eIF2α and p-eIF2α, and quantification of p-eIF2α (n = 3). (**C**) Immunofluorescence analysis and quantification of CHOP (n = 3). (**D**) Immunoblotting analysis of JNK, p-JNK, cleaved caspase-3, and *γ*H2AX, and quantification of p-JNK, cleaved caspase-3, and *γ*H2AX (n = 3). (**E**) TUNEL staining (n = 3). Scale bars in (**A**,**C**,**E**), 10 μm. *** *p* < 0.001.

Our previous study has demonstrated that light exposure activates the PERK/eIF2α/ATF4/CHOP signaling pathway in the neural retina of *Abca4^−/−^Rdh8^−/−^* mice, as compared to C57BL/6J mice [26]. In this study, we investigated the effect of quercetin on this axis in the neural retina of *Abca4^−/−^Rdh8^−/−^* mice upon exposure to light. Immunofluorescence analysis of retinal tissue sections revealed that quercetin significantly reduced protein levels of p-PERK in photoreceptors of light-exposed *Abca4^−/−^Rdh8^−/−^* mice (Figure 5A). Immunoblotting analysis of neural retina extracts showed that quercetin remarkably attenuated p-eIF2α protein levels in *Abca4^−/−^Rdh8^−/−^* mice with light exposure (Figure 5B). Moreover, quercetin markedly inhibited CHOP activation and reduced protein levels of p-JNK, cleaved caspase-3, and γH2AX in the neural retina of *Abca4^−/−^Rdh8^−/−^* mice after exposure to light (Figure 5C,D). The results of TUNEL staining showed a substantial decrease in the number of apoptotic cells in the photoreceptor ONL from quercetin-treated *Abca4^−/−^Rdh8^−/−^* mice with light illumination (Figure 5E). These findings imply that quercetin mitigates light-induced photoreceptor apoptosis in *Abca4^−/−^Rdh8^−/−^* mice via repressing the activation of the PERK/eIF2α/ATF4/CHOP signaling pathway.

## 3. Discussion

Dietary polyphenols are a class of phenolic phytochemicals abundantly found in fruits and vegetables, recognized for their beneficial physiological effects and low toxicity [37]. Among these, quercetin is a notable active compound isolated from numerous plant sources, exhibiting potent anti-inflammatory and antioxidant activities [38]. Quercetin, a flavone, possesses multiple hydroxyl groups that are crucial for its biological activity (Figure 1A). It has been shown to prevent non-alcoholic fatty liver disease (NAFLD) via AMP-activated protein kinase (AMPK)-mediated mitochondrial autophagy [39]. Quercetin is also found to alleviate light-induced retinal degeneration in rats via the AP-1 pathway [40]. Additionally, quercetin reduces respiratory syncytial virus (RSV)-induced lung inflammation by promoting the polarization of alveolar macrophages (AMs) from the pro-inflammatory M1 phenotype to the anti-inflammatory M2 phenotype [41]. However, the protective effects of quercetin against atRAL-mediated retinal degeneration have not yet been studied. This study provided evidence that quercetin was a promising antioxidant candidate for the treatment of atRAL-induced retinal degeneration, including conditions such as dry AMD and STGD1.

Oxidative stress and subsequent apoptosis are critical pathological processes in retinal degeneration [42,43]. Excess accumulation of atRAL enhances ROS generation, which is recognized as one of the most important causes of dry AMD and STGD1 [44,45]. Our findings indicated that quercetin markedly mitigated the toxic effects of atRAL on photoreceptor cells by reducing ROS generation. Previous studies have established that atRAL-induced oxidative stress leads to DNA damage and activation of caspase-3 via the JNK signaling pathway, thereby promoting photoreceptor apoptosis [7]. In the current study, we examined the influence of quercetin on JNK signaling and apoptosis in photoreceptor cells exposed to atRAL. The data demonstrated that quercetin effectively inhibited JNK phosphorylation as well as downstream caspase-3 cleavage and DNA damage in atRAL-loaded photoreceptor cells (Figure 3A,B). Furthermore, TUNEL staining disclosed that quercetin significantly attenuated atRAL-mediated photoreceptor cell apoptosis (Figure 3C).

Oxidative stress can disrupt ER homeostasis, leading to the accumulation of misfolded proteins within its lumen [46]. The presence of misfolded proteins facilitates the dissociation of BiP from PERK, resulting in PERK oligomerization and subsequent phosphorylation [16]. Activated PERK phosphorylates eIF2α, which in turn promotes the activation of ATF4 and CHOP, ultimately culminating in cell apoptosis [25]. ER stress is implicated in a range of ocular diseases, including glaucoma, cataracts, and diabetic retinopathy [47]. Moreover, ER stress drives light-mediated retinal degeneration in an experimental mouse model [48]. Our previous study has demonstrated that ROS visibly localize in the ER of atRAL-treated photoreceptor cells, which activates the PERK/eIF2α/ATF4/CHOP signaling pathway, finally promoting apoptosis [26]. In this work, we presented evidence that quercetin significantly reduced the localization of ROS in the ER of atRAL-treated photoreceptor cells (Figure 1C), thereby inhibiting the activation of the PERK/eIF2α/ATF4/CHOP pathway and consequently suppressing apoptosis (Figure 2 and Figure 3C).

Building on the in vitro antioxidant effects of quercetin, we further explored its in vivo impact. Previous research has revealed that intense light exposure in *Abca4^−/−^Rdh8^−/−^* mice leads to the accumulation of atRAL in the retina, resulting in photoreceptor atrophy and retinal degeneration [7]. Notably, the neural retina of *Abca4^−/−^Rdh8^−/−^* mice subjected to light exposure exhibited remarkable apoptosis and activation of the ER stress-mediated PERK/eIF2α/ATF4/CHOP signaling compared to that of normal C57BL/6J mice [26]. In this study, we established that intraperitoneal administration of quercetin effectively mitigated light-induced retinal damage in *Abca4^−/−^Rdh8^−/−^* mice. Following quercetin treatment, we observed significant restoration of retinal function (Figure 4A,B), notable improvements in the thickness of both the photoreceptor ONL and the overall retinal structure in light-exposed *Abca4^−/−^Rdh8^−/−^* mice (Figure 4C,D), and a marked reduction in fundus punctate lesions (Figure 4E). Furthermore, immunoblotting and immunofluorescence analyses revealed that quercetin significantly attenuated the activation of the PERK/eIF2α/ATF4/CHOP signaling pathway in the neural retina of *Abca4^−/−^Rdh8^−/−^* mice with light illumination (Figure 5A−D). TUNEL staining (Figure 5E) also corroborated the findings that quercetin significantly alleviated light-induced apoptosis in the neural retina of *Abca4^−/−^Rdh8^−/−^* mice.

This study has two limitations. First, although DMSO was used to enhance the solubility of quercetin and facilitate its cellular uptake in both in vitro and in vivo experiments, further optimization of quercetin’s solubility is necessary. Second, while the therapeutic efficacy of quercetin was demonstrated in cellular and animal models, its translational potential requires validation through rigorous clinical trials.

**Figure 6:**
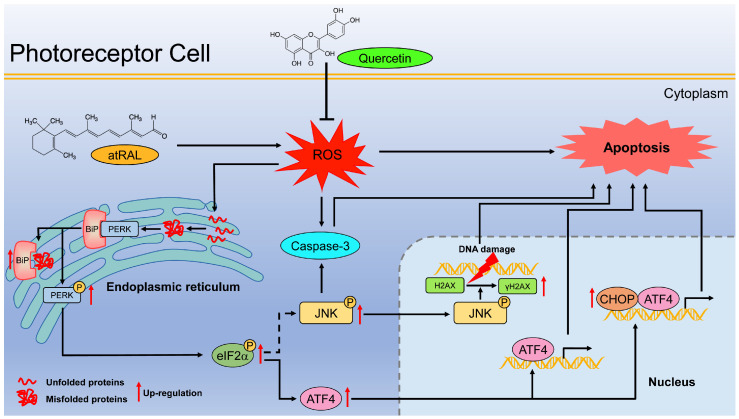
Schematic diagram illustrating the protective effects of quercetin against atRAL-induced apoptosis in photoreceptor cells.

Considered collectively, this study elucidates the protective effect of quercetin on the retina characterized by the atRAL overload and its underlying mechanisms. As depicted in Figure 6, in atRAL-exposed photoreceptor cells, quercetin preserves ER homeostasis by reducing ROS generation, thereby inhibiting photoreceptor apoptosis through modulation of the ER stress-induced PERK/eIF2α/ATF4/CHOP signaling pathway. These findings suggest that quercetin may serve as a potential therapeutic agent for atRAL-associated retinopathies.

## 4. Materials and Methods

### 4.1. Reagents

All reagents used for this study are listed in Table S1.

### 4.2. Animals

The genetic background of *Abca4^−/−^Rdh8^−/−^* mice has been confirmed in a previous study from our laboratory [7]. Animal studies were approved by the Institutional Animal Care and Use Committee of Xiamen University School of Medicine. Four-week-old mice were used to construct the light-induced retinal degeneration model. Briefly, *Abca4^−/−^Rdh8^−/−^* mice were randomly divided into 4 groups (6 mice in each group): a control group, a light-exposed group, a quercetin-treated group, and a light-exposed quercetin-treated group. The mice in the light-exposed group and the light-exposed quercetin-treated group were first dark-adapted for 48 h, and then intraperitoneally injected with DMSO (vehicle) or quercetin (20 mg/kg) for 1 h before light exposure. They then continued to be housed in a dark environment and received daily quercetin or DMSO treatment for 4 days. The control group and the quercetin-treated group only received DMSO or quercetin treatment without light exposure. On the fifth day after light exposure, all mice were further tested.

### 4.3. Cell Culture and Cell Viability

The source and culture conditions of murine photoreceptor cell line 661W have been described in our previous study [7]. Assessment of cell viability was performed by MTS assay [49]. After incubation with graded concentrations (10, 20, and 30 μM) of quercetin for 1 h, 661W cells were treated with 5 μM atRAL for 6 h and then detected using MTS assay in a GO-1510 microplate reader (Thermo Fisher Scientific, Waltham, MA, USA). Note that quercetin was initially dissolved in dimethyl sulfoxide (DMSO) to create a stock solution, which was subsequently diluted to the desired working concentrations in culture medium.

### 4.4. Fluorescence Imaging of ROS and Endoplasmic Reticulum (ER)

H2DCFDA and ER-tracker probes were used to image ROS and ER, respectively. After incubation with 10 μM H2DCFDA, 1 μM ER-Tracker, and 10 μM Hochest 33342 at 37 °C for 20 min, cells were washed three times with PBS and examined using a Zeiss LSM 880 confocal microscope (Zeiss, Jena, Germany). Fluorescence intensity was quantified by ImageJ software (version 2.9.0) and shown as fold changes relative to controls.

### 4.5. Immunofluorescence Staining

The methods of immunofluorescence staining for cells and tissue sections have been described in detail in a previous study [26]. In brief, cells fixed with 4% paraformaldehyde and permeabilized with 0.2% Triton and retinal sections after tissue antigen retrieval were blocked with 2% bovine serum albumin for 1 h, incubated with primary antibodies (1:100 dilution) at 4 °C overnight, and then incubated with Alexa Fluor 594-conjugated fluorescent secondary antibodies (1:200 dilution) for 2 h at room temperature. Finally, the samples were mounted with DAPI and imaged using a Zeiss LSM 880 confocal microscope (Jena, Germany).

### 4.6. TUNEL Staining

Assessment of apoptosis was performed by a TUNEL assay kit [7]. After cells were fixed and permeabilized, TUNEL reagent was used to evaluate cell apoptosis. After a series of steps, including dewaxing with xylene and absolute ethanol, fixation with 4% paraformaldehyde, permeabilization with proteinase K, and re-fixation with 4% paraformaldehyde, retinal tissue sections were evaluated for apoptotic cells using the TUNEL assay. Nuclei were labeled with DAPI. Images were visualized using a Zeiss LSM 880 confocal microscope (Jena, Germany).

### 4.7. Western Blotting

Extracts from cells or neural retina tissues were used for Western blotting as described previously [45]. After electrophoresis and transfer, the membrane was incubated with primary antibodies (1:1000 dilution) overnight at 4 °C, followed by incubation with secondary antibodies (1:2000 dilution) for 2 h at room temperature. Protein bands were subsequently visualized using an imaging system (Bio-Rad, Montreal, QC, Canada).

### 4.8. H&E Staining

H&E staining was performed as previously described [26]. Tissue sections, 3 microns thick, were cut from paraffin-embedded samples and stained with H&E using standard procedures. Images and measurements of the outer nuclear layer (ONL) thickness were obtained using a DM2500 microscope (Leica, Wetzlar, Germany).

### 4.9. Optical Coherence Tomography (OCT) and Fundus Imaging

Mice were anesthetized via intraperitoneal injection of 200 μL of 1% pentobarbital sodium, and pupil dilation was induced using 1% tropicamide. To prevent cataract formation due to dryness, a drop of 0.2% carbomer solution was applied to each eye. Fundus images were captured using a retinal imaging system (Optoprobe; OPIMG-L, Wales, UK). OCT images were acquired with a retinal OCT system (Optoprobe; OPIMG-L, Wales, UK). Retinal thickness was quantified using ImageJ software (version 2.9.0).

### 4.10. Electroretinogram (ERG)

Five days post light exposure, full-field ERG was performed using an Animal Electroretinogram System (Diagnosys LLC, Lowell, MA, USA). All procedures were carried out under dim red-light conditions. Dark-adapted mice were anesthetized via isoflurane inhalation, and pupil dilation was achieved with tropicamide. Scotopic ERG was elicited by a single flash stimulus at an intensity of 1 cd s/m^2^. The responses to brief flashes were analyzed by measuring the amplitudes of a- and b-waves. All ERG recordings were conducted at the same time of day to maintain consistency.

### 4.11. Statistical Analysis

The results are expressed as the mean ± standard deviation (SD) from three independent experiments. Data were analyzed by one-way ANOVA followed by Tukey’s multiple comparisons test using GraphPad Prism software (version 8.0). In all cases, *p*-values below 0.05 were considered statistically significant. ** *p* < 0.01; *** *p* < 0.001.

## Data Availability

The data presented in this study are available on request from the corresponding author.

## Supplementary Materials

The supporting information can be downloaded at: https://www.mdpi.com/article/10.3390/ijms252413624/s1.
