# Supplementary material for: Quercetin Alleviates All-Trans-Retinal-Induced Photoreceptor Apoptosis and Retinal Degeneration by Inhibiting the ER Stress-Related PERK Signaling
Source: Int J Mol Sci. 2024 Dec 19;25(24):13624. doi: 10.3390/ijms252413624 (PMC11727799; doi:10.3390/ijms252413624)

**Supplementary Information**

Quercetin Alleviates All-*Trans*-Retinal-Induced Photoreceptor Apoptosis and Retinal Degeneration by Inhibiting the ER Stress-Related PERK Signaling

Bo Yang^1^, Kunhuan Yang^1^, Ruitong Xi^1^, Jingmeng Chen^2,3^ and Yalin Wu^1,3,^*

1. Fujian Provincial Key Laboratory of Ophthalmology and Visual Science, Fujian Engineering and Research Center of Eye Regenerative Medicine, Eye Institute of Xiamen University, School of Medicine, Xiamen University, Xiamen, Fujian 361102, China
2. School of Medicine, Xiamen University, Xiamen, Fujian 361102, China
3. Shenzhen Research Institute of Xiamen University, Shenzhen, Guangdong 518057, China

*Correspondence: yalinw@xmu.edu.cn (Y. Wu)

**Supplementary Table S1.** **Detailed reagents.**

| **Reagents** | **Catalog** | **Company** |
| --- | --- | --- |
| Quercetin | HY-18085 | MedChemExpress |
| All-*trans*-retinal (atRAL) | R2500 | Sigma-Aldrich |
| Hoechst 33342 | B2261 | Sigma-Aldrich |
| 4′,6-diamidino-2-phenylindole (DAPI) | F6057 | Sigma-Aldrich |
| Dimethyl sulfoxide (DMSO) | D8371 | Solarbio |
| 2',7'-dichlorodihydrofluorescein diacetate (H2DCFDA) | D399 | ThermoFisher Scientific |
| ER-Tracker Red | 40764ES20 | Yeasen |
| Anti-Bip | 3177S | Cell Signaling Technology |
| Anti-p-PERK | 3179S | Cell Signaling Technology |
| Anti-eIF2α | 9722S | Cell Signaling Technology |
| Anti-p-eIF2α | 3398S | Cell Signaling Technology |
| Anti-ATF4 | 11815S | Cell Signaling Technology |
| Anti-CHOP | 5554S | Cell Signaling Technology |
| Anti-PARP | 9542S | Cell Signaling Technology |
| Anti-cleaved caspase 3 | 9661S | Cell Signaling Technology |
| Anti-GAPDH | 5174S | Cell Signaling Technology |
| Anti-**γ**H2AX | 05-636 | ﻿Millipore |
| Alexa Fluor 594-conjugated  donkey anti-rabbit ﻿secondary  antibody | A21207 | ﻿Invitrogen |
| Goat anti-rabbit IgG (H + L) secondary antibody | 31460 | Invitrogen |
| Goat anti-mouse IgG (H + L) secondary antibody | 31430 | Invitrogen |
| ﻿TUNEL assay kit | G3250 | ﻿Promega |
| MTS assay kit | G3580 | Promega |

**Supplementary Figure S1. Unprocessed western blots.**

Boxes in *red* indicate selected Western blot results.

**Fig. 2A**


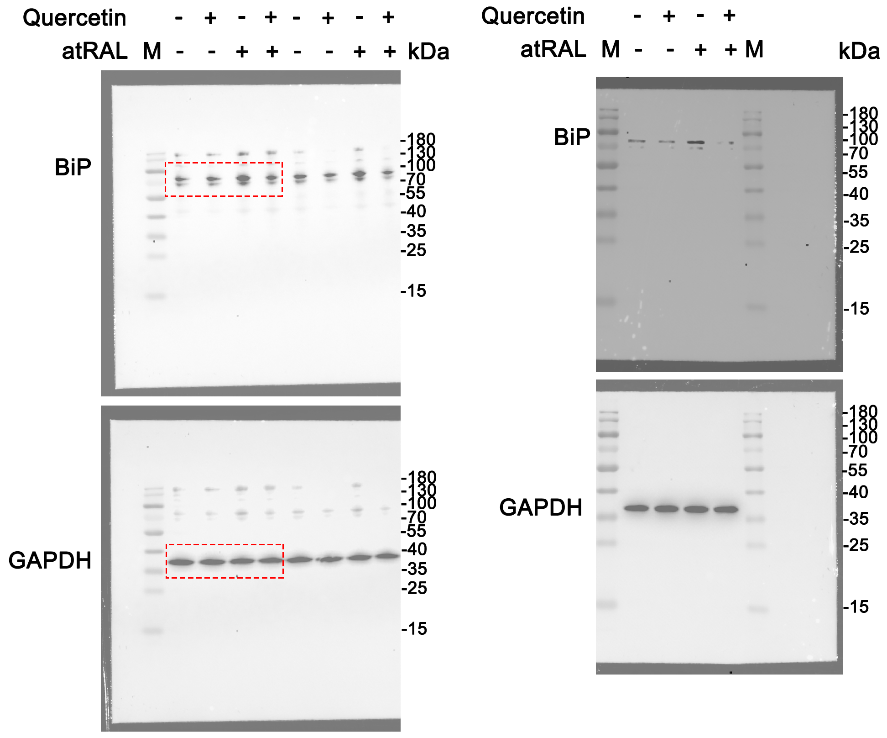


**Fig. 2C**


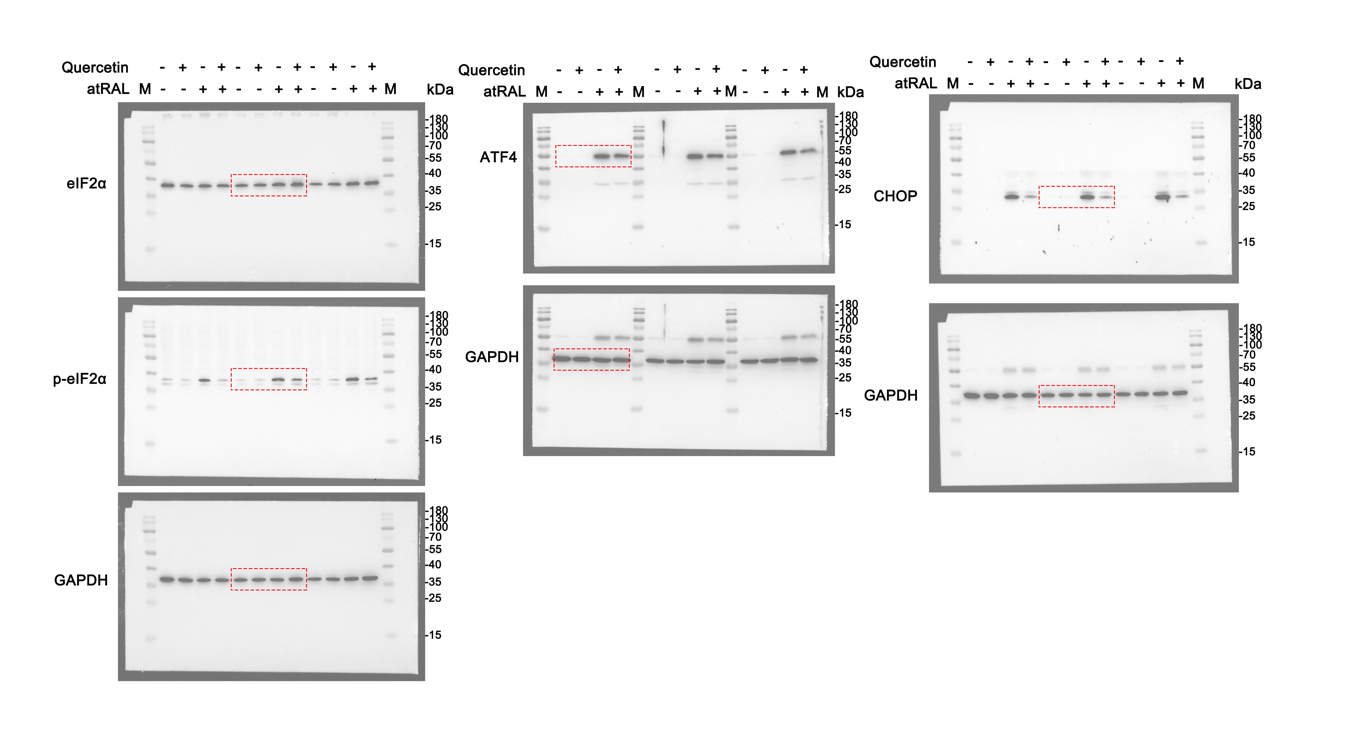


**Fig. 3A**


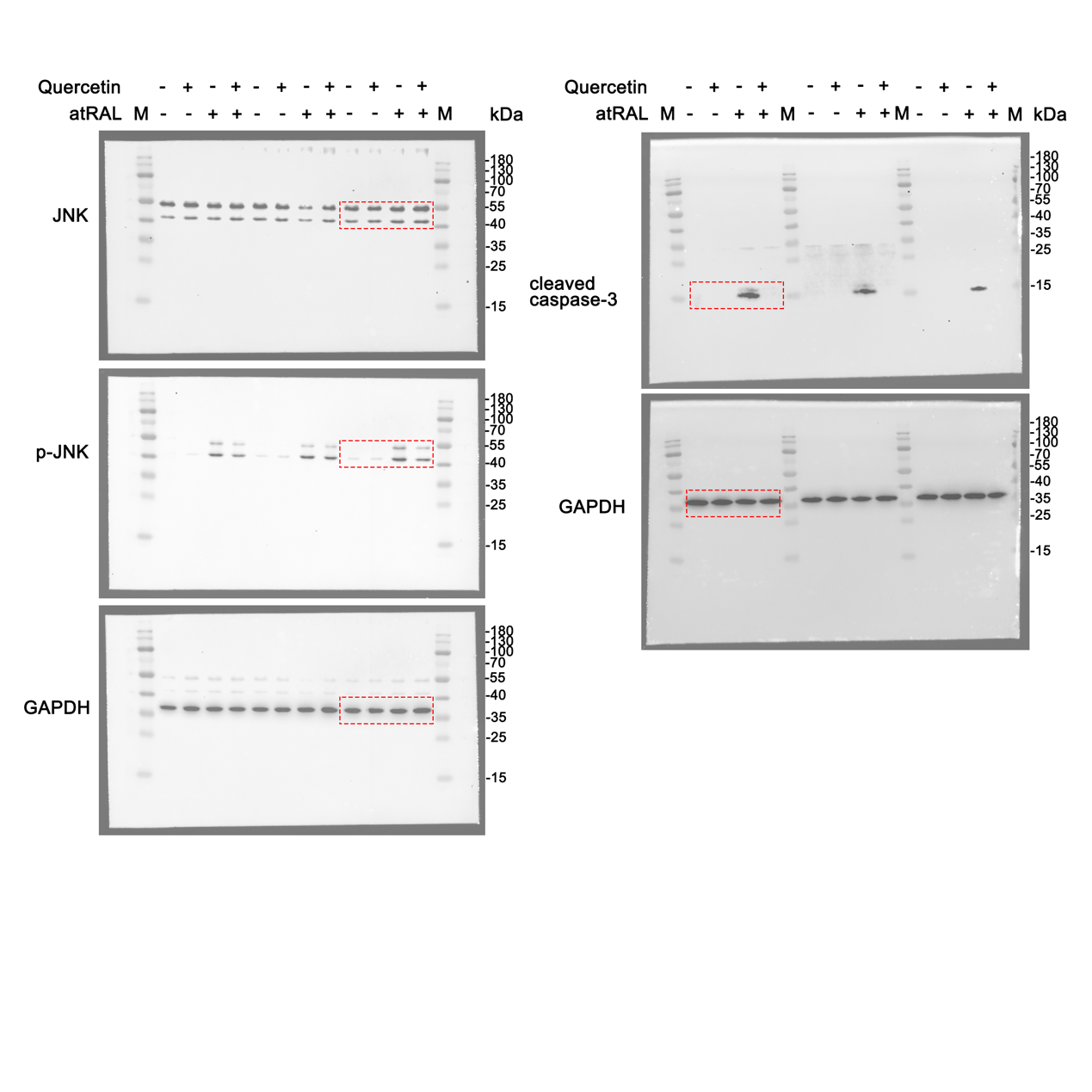


**Fig. 3B**


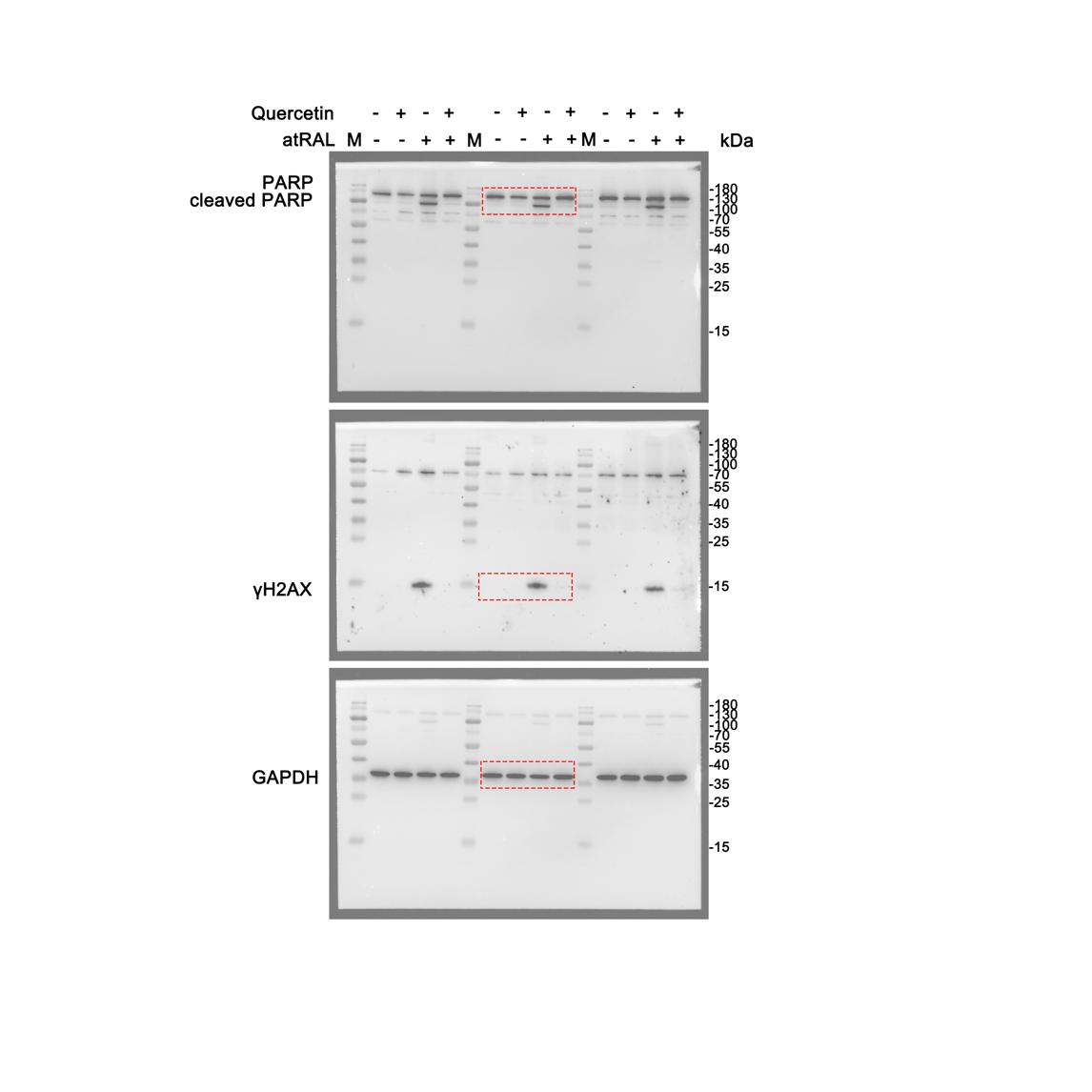


**Fig. 5B**


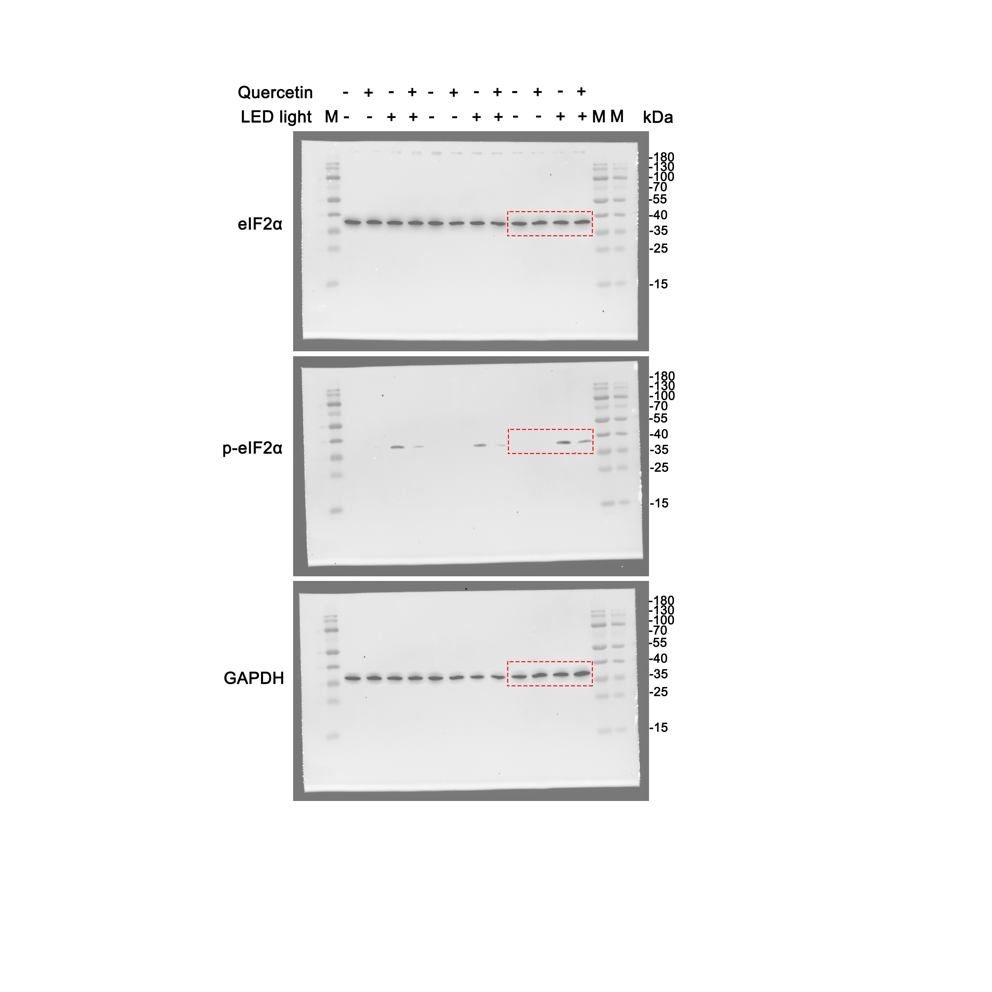


**Fig. 5D**


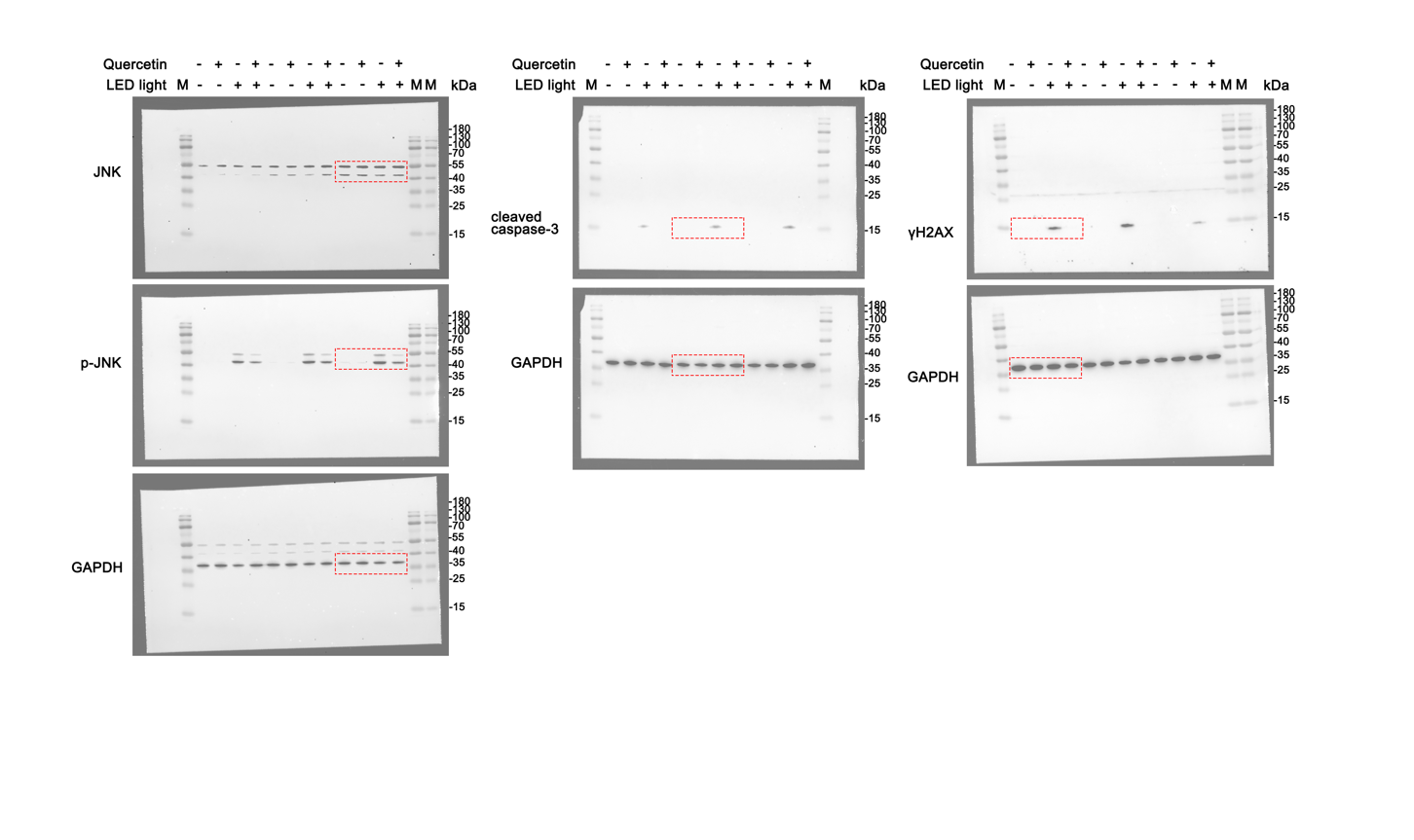

Supplement: Supplementary file 1 [file ijms-25-13624-s001.zip › IJMS-SI revised 1.docx]
